# Efficacy and safety of high-dose ilaprazole-amoxicillin dual therapy for *Helicobacter pylori* eradication: a prospective, single-center, randomized trial

**DOI:** 10.3389/fphar.2023.1272744

**Published:** 2023-11-07

**Authors:** Jianping Cheng, Chanjuan Fan, Kun Huang, Lili Zhai, Hui Wang, Dongling Xie, Yong Cai, Zhen Li, Qixuan Bai, Pan Wang, Haiou Ding

**Affiliations:** Department of Gastroenterology and Oncology, Civil Aviation General Hospital, Beijing, China

**Keywords:** *Helicobacter pylori*, ilaprazole, proton‐pump inhibitor, dual therapy, eradication

## Abstract

**Background:** Until now, there have been no randomized controlled trials directly evaluating the efficacy of high-dose ilaprazole-amoxicillin dual therapy (HT) in comparison to other standard treatments for *H. pylori* (*Helicobacter pylori*) infection. This study aimed to compare the effectiveness and safety of HT with bismuth quadruple therapy (BQT) as an initial treatment for *H. pylori*.

**Methods:** This single-center, prospective, randomized clinical controlled trial recruited 225 consecutive patients. They were assigned to either HT group (ilaprazole, 10 mg, twice daily; amoxicillin 1,000 mg, three times daily) or BQT group (compound bismuth aluminate granules, 2.6 g, three times daily; ilaprazole, 5 mg, twice daily; amoxicillin, 1,000 mg, twice daily; clarithromycin, 500 mg, twice daily) for 14 days. The ^13^C-urea breath test assessed eradication success 4 weeks after treatment. The primary outcome focused on the eradication rate, with secondary outcomes including safety and compliance.

**Results:** From February 2023 to March 2023, 228 subjects were screened, and 225 were randomized. The HT and BQT groups showed eradication rates of 76.3% and 61.3% (*p* = 0.015) both by intention-to-treat (ITT) analysis and per-protocol (PP) analysis. HT was associated with fewer adverse events than BQT (27.2% vs. 81.8%, *p* = 0.002). The most commonly reported adverse events was bitter taste of mouth (3.5% vs. 60.4%, *p* < 0.001). There was no significant difference in compliance between the two groups (89.5% vs. 92.8%, *p* = 0.264).

**Conclusion:** The 14-day HT treatment demonstrates better efficacy in *H. pylori* eradication treatment and improved safety and compliance compared to BQT. The results provide supporting evidence for 14-day HT can be potentially considered as a first-line regimen for empirical treatment.

**Clinical Trial Registration:** https://www.chictr.org.cn/showproj.html?proj=186562, identifier ChiCTR2200066284

## 1 Introduction

*Helicobacter pylori* (*Helicobacter pylori*) is a persistent bacterial infection that affects more than half of the global human population (Suzuki et al., 2020). Although the prevalence of *H. pylori* infection is declining in the developed countries, it remains a substantial cause of global morbidity and mortality (Sabari et al., 2017). *Helicobacter pylori* significantly contributes to a range of gastroduodenal diseases, including not only chronic gastritis and peptic ulcer disease, but also more serious conditions like mucosa-associated lymphoid tissue lymphoma and gastric cancer (Devi et al., 2016). In recognition of its carcinogenic potential, both the World Health Organization (WHO) in 1994 and the United States Department of Health and Human Services (HHS) in 2022 classified *H. pylori* as a group 1 or definite carcinogen (Zhu et al., 2017; Duan et al., 2023). The eradication of *H. pylori* infection has, therefore, become a pressing public health concern worldwide.

In more recent years, the eradication rate of standard *H. pylori* therapies has declined due to the increasing resistance to antibiotics, especially to clarithromycin. The eradication rate with standard triple therapy has fallen below 80%, largely due to bacterial factors, patient compliance, genetic polymorphisms in CYP2C19, and, most notably, antibiotics resistance (Kuo et al., 2019; Chen et al., 2020). A systematic review of 65 countries revealed primary resistance rates to commonly used antibiotics, such as clarithromycin, metronidazole, and levofloxacin, exceeding 15% in many regions (Gisbert, 2020). Therefore, recent international guidelines have recommended either bismuth or non-bismuth quadruple therapies, administered for 10–14 days as a first-line eradication regimen, particularly in areas with high clarithromycin resistance (Chey et al., 2017; Malfertheiner et al., 2017; Zhou et al., 2022). Nonetheless, these regimens have several challenges, including drug-related side effects, treatment costs, and reduced patient compliance, limiting their routine use in clinical practice (Lin et al., 2022). Moreover, reliance on multiple antibiotics in treating *H. pylori* speeds up the advent of antimicrobial resistance, undermining their future effectiveness. Consequently, there is a growing need for innovative alternative regimens that can manage antimicrobial resistance and ensure optimal eradication rates while limiting antibiotics use.

High-dose dual therapy, comprising of a proton-pump inhibitor (PPI) and amoxicillin, offers a simplified approach to *H. pylori* treatment. With its single antibiotic therapy, effective acid suppression, and low antibiotic resistance, high-dose dual therapy is receiving significant attention (Bi et al., 2022). Studies have demonstrated that the eradication rate of high-dose dual therapy in early *H. pylori* treatment is comparable to or even better than traditional PPI-based triple or bismuth quadruple therapies, with the added benefit of fewer side effects (Zhu et al., 2020; Yun et al., 2021). Ilaprazole, a new generation PPI, consistently and effectively inhibits gastric acid secretion, making it an attractive option for high-dose dual therapy (Wang et al., 2019). Furthermore, ilaprazole effectively overcomes the CYP2C19-related limitations associated with other PPIs, the plasma area under curve and peak concentration of ilaprazole are not influenced by CYP2C19 genetic polymorphisms (Pu et al., 2018). However, the effectiveness of this dual therapy for *H. pylori* eradication has not yet been explored, and no randomized studies have evaluated the efficacy of high-dose dual therapy for *H. pylori* eradication.

In this study, we aimed to evaluate the efficacy, safety, and tolerability of high-dose ilaprazole-amoxicillin dual therapy (HT) and compare it with bismuth-containing quadruple therapy (BQT) as alternative treatments for *H. pylori* infection. The findings of this study offer additional scientific evidence supporting HT as an acceptable regimen for first-line *H. pylori* treatment.

## 2 Material and methods

### 2.1 Study design and ethical approval

This was a prospective, single-center, non-inferiority, randomized controlled trial. The primary objective was to confirm that HT is not inferior to BQT as a first-line treatment for *H. pylori* infection. This study was carried out in line with the Declaration of Helsinki and adhered to the guidelines of the Consolidated Standards of Reporting Trials (CONSORT). The study protocol received approval from the Institutional Ethics Board of the Civil Aviation General Hospital, Beijing, China (No. 2022-L-K-60). The trial was registered in the Chinese Clinical Trials Registration (www.chictr.org.cn) with the registration number ChiCTR2200066284.

### 2.2 Participants

Between February 2023 and March 2023, consecutive outpatients confirmed to have *H. pylori* infection via the ^13^C-urea breath test (UBT) were eligible for enrollment. A total of 225 eligible patients were enrolled in outpatient clinics. In this study, the eligibility criteria for patient inclusion were as follows: 1) Age between 18 and 70 years old; 2) Tested positive for *H. pylori* infection by ^13^C-UBT; 3) Had not received any prior *H. pylori* eradication treatment; 4) Signed the informed consent form. Patients were excluded from the study if they met any of the following criteria: 1) Previously received standard *H. pylori* therapy; 2) Had taken antibiotics, bismuth, or PPIs prior to the start of treatment; 3) Had an allergy or contraindication to the drugs used in the trial; 4) Suffered from a serious primary disease; 5) Had clinically significant liver or kidney insufficiency; 6) Had a history of alcohol abuse; 7) Were pregnant or lactating women.

### 2.3 Randomization and intervention

The patients were randomly assigned to the HT or the BQT group in a 1:1 ratio using block randomization (blocks of 4) with the sequentially numbered, opaque sealed envelope technique (Paavola et al., 2021). The HT consisted of 10 mg ilaprazole (Livzon Pharmaceutical Group, Zhuhai, China) twice daily and 1,000 mg amoxicillin three times daily for 14 days. The BQT consisted of 5 mg ilaprazole twice daily, 2.6 g compound bismuth aluminate granules (200 mg of bismuth) three times daily, 1,000 mg amoxicillin twice daily and 500 mg clarithromycin twice daily for 14 days.

### 2.4 Study outcomes

The primary endpoint was the *H. pylori* eradication rate in the HT and BQT groups, which was assessed by ^13^C-UBT at 4–6 weeks after the end of the treatment. The primary analysis for this study utilized both intention-to-treat (ITT) and per-protocol (PP) analyses. In the ITT analysis, all randomized patients were included, regardless of their adherence to the treatment or undergoing ^13^C-UBT. However, for patients who were lost to follow-up or did not undergo ^13^C-UBT, they were considered as treatment failures in the ITT analysis. On the other hand, the PP analysis only included patients who achieved at least 80% compliance to the study medication and underwent ^13^C-UBT. Compliance was determined through pill count and taking 80% or more of the study medication was defined as good compliance in this analysis.

In this study, the secondary endpoints focused on the incidence and severity of adverse events (AEs). A specific questionnaire form was given to patients, who were instructed to fill it out for 14 days from the beginning of the therapy, specifically noting any AEs induced by the study drugs. Whenever patients reported any AE in the questionnaire form, the investigators would inquire about them further and assess the severity using a grading system ranging from 1 to 5. This grading system was based on the National Cancer Institute (NCI) Common Terminology Criteria for Adverse Events (NCI-CTCAE) Version 5.0. The outcomes in this study were not altered or modified after the trial began, ensuring the integrity of the data collected.

### 2.5 Sample size estimation and statistical analysis

Based on a previous study, the 14-day dual therapy had an 88% eradication rate, while the 14-day quadruple therapy achieved an 84% rate (Niu et al., 2022). Statistically, a non-inferiority margin of −10% was the recommended level in a non-inferiority anti-infective trial and in *H. pylori* treatment trials. Assuming a power of 80% and an alpha of 0.025 (one-sided), at least 194 patients (97 patients in each group) would be required. Based on an anticipated follow-up loss of 15%, a sample size of 228 patients (114 patients in each group) was planned.

Statistical analyses to identify prediction factors were conducted using SPSS 26.0 (SPSS, Chicago). To assess the comparative non-inferiority of the two groups, a two-sided 95% confidence interval (CI) and hypothesis testing (one-sided μ-test) were derived. Differences between the groups were analyzed using Pearson’s χ^2^ test for categorical variables and Student’s t-test for continuous variables. In this analysis, all *p*-values were considered two-sided, except for the test of non-inferiority. A *p*-value less than 0.05 was considered statistically significant, indicating a significant difference between the groups. The test of non-inferiority has used a one-sided *p*-value threshold to determine whether one group’s outcome was not significantly worse than the other group.

## 3 Results

### 3.1 Patient enrolment and baseline characteristics

**FIGURE 1:**
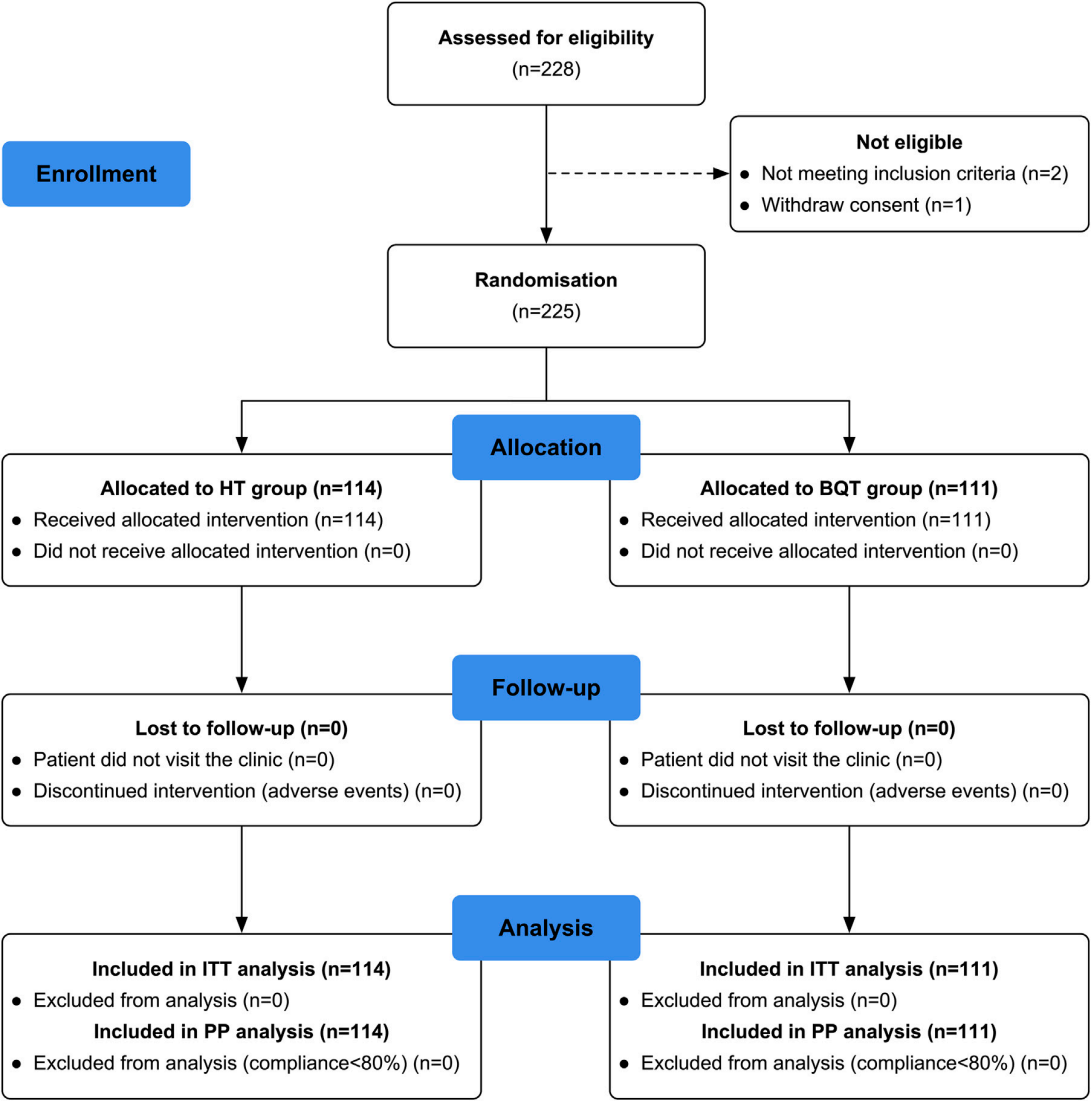
Flow chart of patient enrolment and study design. ITT, intention-to-treat; PP, per-protocol; HT, high-dose ilaprazole-amoxicillin dual therapy; BQT, bismuth-containing quadruple therapy.

In this study, the flow chart of patient enrollment and allocation is shown in Figure 1. A total of 228 patients were initially assessed for eligibility, out of which 225 patients were recruited for the study. These patients were then randomly divided into two groups: the HT group with 114 patients and the BQT group with 111 patients. Baseline demographic data and clinical characteristics were compared between the two groups and no significant differences were found (Table 1). It is worth noting that there were no patients lost to follow-up or excluded from the ^13^C-UBT procedure, indicating a high compliance and completeness of the study.

**TABLE 1:** Baseline characteristics of the study patients.

Characteristic	HT group (n = 114)	BQT group (n = 111)	Statistics	*p*-value
Age, y	40.96 ± 11.93	41.74 ± 12.10	−0.488	0.626
Age group				
<50	87 (76.3)	80 (72.1)	0.529	0.467
≥50	27 (23.7)	31 (27.9)		
Sex				
Male	47 (41.2)	52 (46.8)	0.721	0.396
Female	67 (58.8)	59 (53.2)		
BMI (kg/m^2^)	23.78 ± 4.42	24.08 ± 4.51	−0.511	0.610
BMI group				
<24	68 (59.6)	61 (55.0)	0.507	0.477
≥24	46 (40.4)	50 (45.0)		
Alcohol drinking			0.126	0.723
No	109 (95.6)	105 (94.6)		
Yes	5 (4.4)	6 (5.4)		
Smoking			0.465	0.495
No	96 (84.2)	97 (87.4)		
Yes	18 (15.8)	14 (12.6)		
Disease duration, m			0.370	0.543
≤1	79 (69.3)	81 (73.0)		
>1	35 (30.7)	30 (27.0)		
Marital status			0.037	0.847
Married	85 (74.6)	84 (75.7)		
Single or divorced or widowed	29 (25.4)	27 (24.3)		
Past medical history			0.075	0.785
No	99 (86.8)	95 (85.6)		
Yes	15 (13.2)	16 (14.4)		
Concomitant medication			1.996	0.158
No	106 (93.0)	97 (87.4)		
Yes	8 (7.0)	14 (12.6)		
Comorbidity				
Diabetes	7 (6.1)	6 (5.4)	0.056	0.813
Hypertension	6 (5.3)	9 (8.1)	0.732	0.392
DOB of^13^C-UBT, median (IQR)	21.45 (12.48,39.13)	18.80 (8.10,32.30)	−0.967	0.334

Categorical data are expressed as number of patients and percentage in parentheses; continuous data are presented as mean ± standard deviation (SD).

Abbreviations: BMI, body mass index; IQR, interquartile range; DOB, differences over baseline; HT, high-dose ilaprazole-amoxicillin dual therapy; BQT, bismuth-containing quadruple therapy.

### 3.2 *Helicobacter pylori* eradication rates

The successful *H. pylori* eradication rates for each therapy are shown in Table 2. In both the ITT and PP analyses, the HT group exhibited an eradication rate of 76.3%, whereas the BQT group showed a rate of 61.3%. A significant difference was observed between the two groups (*p* = 0.015). The *p*-value for non-inferiority was 0.001 in both ITT and PP analyses (Table 2). These findings indicated that HT was not inferior to BQT in terms of *H. pylori* eradication.

**TABLE 2:** Eradication rates of each therapy group.

Analysis	HT group	BQT group	χ^2^	*p*-Value for difference^a^	*p*-Value for non-inferiority^b^
ITT	76.3 (87/114)	61.3 (68/111)	5.947	0.015	0.001
PP	76.3 (87/114)	61.3 (68/111)	5.947	0.015	0.001

Data are expressed as percentage with number of patients included in parentheses.

^a^ The *p* values were obtained from two-sided comparisons of difference between the HT, group and BQT, group.

^b^ The *p* values were obtained from one-sided test comparisons of non-inferiority between the HT, group and BQT, group.

Abbreviations: ITT, intention-to-treat; PP, per-protocol; CI, confidence interval; HT, high-dose ilaprazole-amoxicillin dual therapy; BQT, bismuth-containing quadruple therapy.

### 3.3 Factors affecting the efficacy rates

According to the analysis in the PP population (Table 3), the success of the two regimens, HT group and BQT group, was influenced by certain factors. These factors include sex, body mass index, and disease duration. Disease duration was defined as the duration of patients self-reported symptoms (epigastric pain, gastric acid reflux, belching, abdominal bloating and heartburn) when outpatient visit. In the HT group, the eradication rate for females was 79.1% (53/67), while in the BQT group, it was 61.0% (36/59) (*p* = 0.026). For individuals with a body mass index of 24 kg/m^2^ or higher, the HT group had an eradication rate of 80.4% (37/46), compared to 62.0% (31/50) in the BQT group (*p* = 0.047). Moreover, in patients with a disease duration time longer than 1 month, the eradication rate in the HT group was 80.0% (28/35), while it was 43.3% (13/30) in the BQT group (*p* = 0.002). On the other hand, age and marital status did not significantly affect the efficacy of both regimens (*p* > 0.05). These findings suggest that factors such as sex, body mass index, and disease duration time may play a role in determining the success of the two regimens in the PP population.

**TABLE 3:** Factors affecting eradication rates of each therapy group.

Eradication rate in subgroups	HT group (n = 114)	BQT group (n = 111)	χ^2^	*p*-value
Sex				
Male	72.3 (34/47)	61.5 (32/52)	1.296	0.255
Female	79.1 (53/67)	61.0 (36/59)	4.948	0.026
Age, y				
<50	74.7 (65/87)	61.3 (49/80)	3.487	0.062
≥50	81.5 (22/27)	61.3 (19/31)	2.840	0.092
BMI group (kg/m^2^)				
<24	73.5 (50/68)	60.7 (37/61)	2.427	0.119
≥24	80.4 (37/46)	62.0 (31/50)	3.941	0.047
Marital status				
Married	80.0 (68/85)	61.9 (52/84)	6.719	0.010
Single or divorced or widowed	65.5 (19/29)	59.3 (16/27)	0.234	0.629
Disease duration, m				
≤1	74.7 (59/79)	67.9 (55/81)	0.898	0.343
>1	80.0 (28/35)	43.3 (13/30)	9.325	0.002

Data are expressed as percentage with number of patients included in parentheses. Disease duration is defined as the duration of patients self-reported symptoms (epigastric pain, gastric acid reflux, belching, abdominal bloating and heartburn) when outpatient visit.

Abbreviations: HT, high-dose ilaprazole-amoxicillin dual therapy; BQT, bismuth-containing quadruple therapy.

### 3.4 Compliance and adverse events

The drug-induced AEs experienced by the patients during the 14-day treatment mainly included dizziness, skin rash, abdominal pain, abdominal bloating, nausea, diarrhea, constipation, and bitter taste of mouth (Table 4). In the HT group, the frequency of AEs was 27.2% (31/114) of patients, while in the BQT group, the frequency of AEs was 81.8% (90/111) of patients (*p* = 0.002). The AEs were all mild and moderate. The frequency of bitter taste of mouth (*p* < 0.001) was significantly lower in the HT group compared with the BQT group. The compliance rates were 89.5% (102/114) in the HT group and 92.8% (103/111) in the BQT group, with no significant difference between the two groups (*p* > 0.05).

**TABLE 4:** Drug-induced adverse effects and patient adherence of each therapy group.

Variable	HT group (n = 114)	BQT group (n = 111)	χ^2^	*p*-value
Total	31 (27.2)	90 (81.8)	9.325	0.002
Adverse events grade				
Mild	25	85		
Moderate	6	5		
Severe	0	0		
Adverse events				
Dizziness	1 (0.9)	1 (0.9)	-	-
Skin rash	4 (3.5)	1 (0.9)	-	-
Abdominal pain	6 (5.3)	5 (4.5)	0.070	0.792
Abdominal bloating	10 (8.8)	9 (8.1)	0.032	0.858
Nausea	2 (1.8)	1 (0.9)	-	-
Diarrhea	3 (2.6)	2 (1.8)	-	-
Constipation	1 (0.9)	4 (3.6)	-	-
Bitter taste of mouth	4 (3.5)	67 (60.4)	84.162	<0.001
Treatment compliance	102 (89.5)	103 (92.8)	1.250	0.264

Data are expressed as number of patients with percentage included in parentheses. Adverse events were assessed in the per-protocol (PP) population. Treatment compliance was indicative of patients who took at least 80% of study drugs.

Abbreviations: HT, high-dose ilaprazole-amoxicillin dual therapy; BQT, bismuth-containing quadruple therapy.

## 4 Discussion

In this study, we assessed the effectiveness and safety of a 14-day high-dose dual therapy combining ilaprazole and amoxicillin as a first-line treatment for eradicating *H. pylori* infection. To the best of our knowledge, this is the first randomized controlled study of the use of ilaprazole 10 mg twice daily and amoxicillin 1,000 mg three times daily in a 14-day dual therapy to eradicate *H. pylori* infection. Our findings demonstrated that the eradication rate of HT was significantly superior to BQT (76.3% vs. 61.3%, *p* = 0.015), which indicated that 14-day HT is an acceptable alternative therapy for *H. pylori* infection treatment.

Currently, the increasing resistance rates of *H. pylori* to clarithromycin and metronidazole have resulted in decreased efficacy of the standard triple therapy (PPI, clarithromycin, amoxicillin, or metronidazole) for eradication. As a result, four-drug combination therapies, such as BQT or concomitant quadruple therapy, are now recommended as first-line treatments for *H. pylori* (Graham and Fischbach, 2010; Argueta et al., 2021; Lee et al., 2022). The fifth Chinese national consensus report on the management of *H. pylori* infection recommends seven eradication regimens for empirical eradication therapy, which involve the use of six different antibiotics (Hu et al., 2019). Indeed, recent studies conducted in China have reported high prevalence rates of resistance among *H. pylori* strains to clarithromycin (20%–50%), metronidazole (40%–70%), and levofloxacin (20%–50%) (Kong et al., 2020). In contrast, resistance rates for amoxicillin (0%–5%), tetracycline (0%–5%), and furazolidone (0%–1%) have remained relatively low and stable over time (Liu et al., 2018). Notably, tetracycline- and furazolidone-based BQT has achieved an 85% eradication rate among patients who have previously failed eradication attempts in China (Delchier et al., 2014). However, the potential adverse drug reactions and limited accessibility of tetracycline and furazolidone hinder their widespread adoption for *H. pylori* treatment. Our study demonstrated that the HT treatment, which combines ilaprazole and amoxicillin, achieved a higher eradication rate when compared to BQT. Therefore, it can be considered a viable and effective alternative for the treatment of *H. pylori*.

PPIs are the most effective agents in suppressing gastric acid secretion and are widely used in the treatment of gastroesophageal reflux and peptic ulcer diseases, as well as in *H. pylori* eradication therapy (Katz et al., 2022). Despite their prevalent usage, PPIs present challenges like individual variability of CYP2C19 polymorphisms, short action duration, and compromised acid inhibition during nighttime (Scarpignato et al., 2016). Ilaprazole is a novel PPI that is developed by Livzon Pharmaceutical Co., Ltd. (Zhuhai, China). Unlike other PPIs, ilaprazole is primarily metabolized by CYP3A and less by CYP2C19. Therefore, the pharmacokinetics and pharmacodynamics of ilaprazole are not significantly affected by CYP2C19 polymorphism (Cho et al., 2012). Studies have shown that ilaprazole reaches maximal plasma concentration between 3.4 and 3.7 h and has an elimination half-life ranging between 8.1 and 10.1 h, which are better results compared to other available PPIs (de Bortoli et al., 2013). Likewise, vonoprazan, a new potassium-competitive acid blocker, has an elimination half-life of up to 9 h (Sakurai et al., 2015). Notably, both ilaprazole and vonoprazan promote the healing of gastric mucosa by inhibiting gastric acids and maintaining a pH greater than 4 for protracted durations (Yang et al., 2022). Taken together, the combination of strong gastric acid suppression and the maintenance of a high intra-gastric pH are likely contributing factors to the high eradication rate observed with ilaprazole-based dual therapy. In this study, we demonstrated that HT achieved a 76.3% eradication rate in the PP analysis and was shown to be non-inferior to BQT. Therefore, HT could be considered an alternative regimen for first-line *H. pylori* treatment.

The dual therapy consisting of PPI and amoxicillin was first reported in the 1990s as a first-line treatment against *H. pylori* infection (Koizumi et al., 1998). Despite enormous effort by clinicians and researchers, there is no consistent evidence of its effectiveness (Unge et al., 1989; Koizumi et al., 1998; Wong et al., 2000; Yang et al., 2019; Yu et al., 2019). Low-dose and infrequent dual therapy with amoxicillin (2.0 g/day or less) and PPI (twice/day or less) has demonstrated inadequate eradication rates (Wong et al., 2000). However, high-dose and frequency dual therapy, involving the administration of amoxicillin (≥2.0 g/day) and PPI (at least twice daily) for 14 days, has demonstrated greater efficacy as a first-line treatment (Suzuki et al., 2020). *Helicobacter pylori* is highly sensitive to amoxicillin at a pH higher than 6, so the continuous use of PPI to inhibit gastric acid is crucial for successful eradication (Labenz, 2001). Additionally, the efficacy of amoxicillin depends on pH, with higher stability and antibacterial effects observed as the pH value increases (Sugimoto et al., 2012). Moreover, amoxicillin functions as a time-dependent antibiotic, requiring prolonged time and higher plasma concentrations to achieve optimal bactericidal effects at an intragastric pH of 5.5 or higher (Denisenko et al., 2018). Consequently, high-dose and high-frequency administration of PPI plus amoxicillin dual therapy is essential for efficacy. Global consensus statements recommend a twice-daily dose of PPIs to maintain gastric acid suppression. Furthermore, these statements suggest that *H. pylori* eradication rates may not be affected by the CYP2C19 genotype (Malfertheiner et al., 2017). The success of a dual therapy regimen largely depends on maintaining a near-neutral pH in the stomach, making *H. pylori* more susceptible to amoxicillin. Ilaprazole, known for its potent acid secretion inhibitory effect compared to other PPIs, is anticipated to be more effective when paired with amoxicillin for *H. pylori* eradication. Since 2022, ilaprazole has been used in dual therapy, resulting in a satisfactory eradication rate. The 14-day HT therapy, comprising of 5 mg ilaprazole twice daily and 750 mg amoxicillin four times daily, achieved *H. pylori* eradication rates of 88% in the ITT analysis and 93% in the PP analysis (Niu et al., 2022). Higher eradication rates were observed with 14-day high-dose ilaprazole-amoxicillin dual therapy, constituting 5.0 mg ilaprazole twice daily and 1 g amoxicillin thrice daily, which resulted in *H. pylori* eradication rates of 92.1% in the ITT analysis and 94.9% in the PP analysis (Zhang et al., 2023). However, the eradication rate of our *H. pylori* eradication regimen did not achieve the clinically sufficient eradication rate of 90%. The 2022 Chinese National Clinical Practice Guideline on *H. pylori* eradication treatment, developed by the Chinese Society of Gastroenterology, states that the average eradication rate for bismuth-containing quadruple therapy stands at 81.3%, and 85.3% for dual therapy (Zhou et al., 2022). Meanwhile, a retrospective study conducted by our team involving over 3,700 patients, showed an eradication rate between 54.1% and 71.6% for bismuth-containing quadruple therapy. Furthermore, the discrepancy in patient numbers and regional antibiotic resistance rates between our study and the aforementioned literature may have contributed to differences in eradication rates. It is well-known that patient characteristics and regional variations in antibiotic resistance patterns play a significant role in the success of *H. pylori* eradication. Until now, it is unclear, however, if 5 mg ilaprazole can maintain the stable acid suppression required for *H. pylori* eradication. By demonstrating the efficacy of the 10 mg ilaprazole dosing regimen, our study fills this gap and offers a reliable foundation for clinicians to select the appropriate dosage of ilaprazole.

High-dose PPI-amoxicillin dual therapy offers several benefits in *H. pylori* therapy, including simplified dosing regimens, increased drug effectiveness, and reduced unnecessary drug use. This approach addresses the global priority of *H. pylori* antimicrobial resistance by decreasing irrational antibiotic use and lowering adverse effects during treatment (Boltin et al., 2019). Due to increasing rates of resistance to clarithromycin and other antibiotic families, international guidelines now advise against their empirical use for *H. pylori* infection. Consequently, it is crucial to re-evaluate treatment strategies and ensure the appropriate use of antibiotics (Arslan et al., 2017). Monitoring of susceptibility of *H. pylori* to antibiotics is essential for selecting effective therapy. However, routine antimicrobial susceptibility testing is not widely conducted due to its invasiveness, limited availability of culture facilities, and cost concerns (Suzuki et al., 2020). The HT regimen offers an alternative for *H. pylori* eradication treatment amid increasing antimicrobial resistance. We have demonstrated that HT achieves satisfactory eradication rates. As it utilizes a single antibiotic, amoxicillin, and does not involve clarithromycin, and given that *H. pylori* exhibits low resistance to amoxicillin, we recommend HT as a primary empirical treatment for *H. pylori*. Therapies based on susceptibility involving multiple antibiotics should be reserved for rescue treatment when HT proves ineffective.

However, this study has several limitations. Firstly, the trial was conducted in Beijing, China, with the majority of participants coming from the local and surrounding regions. Therefore, further studies are needed to verify the applicability of HT in other regions within China or other countries. Secondly, the protocol for this trial was selected empirically, based on previous studies and considerations of compliance. There is a need for further optimization of the medication method involving amoxicillin and PPIs. Lastly, important factors such as CYP2C19 polymorphisms, *H. pylori* strain antimicrobial resistance, and intragastric pH during eradication therapy were not assessed in this study. Future evaluations examining these factors and their impact on eradication efficacy are warranted.

## 5 Conclusion

In conclusion, our findings demonstrate that a 14-day HT treatment is non-inferior to BQT, showing fewer adverse effects and good treatment compliance. This study suggests that HT can serve as a safe and effective alternative for *H. pylori* treatment.

## Data Availability

The original contributions presented in the study are included in the article/Supplementary Material, further inquiries can be directed to the corresponding author.
